# Comparison of proactive and conventional treatment of anastomotic leakage in rectal cancer surgery: a multicentre retrospective cohort series

**DOI:** 10.1007/s10151-023-02808-z

**Published:** 2023-05-22

**Authors:** K. Talboom, N. G. Greijdanus, N. Brinkman, R. D. Blok, S. X. Roodbeen, C. Y. Ponsioen, P. J. Tanis, W. A. Bemelman, C. Cunningham, F. B. de Lacy, Roel Hompes

**Affiliations:** 1 Department of Surgery, Amsterdam UMC, Location AMC, Meibergdreef 9, 1105 AZ Amsterdam, The Netherlands; 2 Department of Gastro-Enterology, Amsterdam UMC, Location AMC, Amsterdam, The Netherlands; 3 Department of Surgery, Amsterdam UMC, Location VUmc, Amsterdam, The Netherlands; 4 Department of Colorectal Surgery, Churchill Hospital, Oxford University Hospitals NHS Foundation Trust, Oxford, UK; 5 Department of Gastrointestinal Surgery, Hospital Clinic of Barcelona, University of Barcelona, Barcelona, Spain

**Keywords:** Rectal cancer, Low anterior resection, Anastomotic leakage, Anastomotic salvage

## Abstract

**Purpose:**

Comparative studies on efficacy of treatment strategies for anastomotic leakage (AL) after low anterior resection (LAR) are almost non-existent. This study aimed to compare different proactive and conservative treatment approaches for AL after LAR.

**Methods:**

This retrospective cohort study included all patients with AL after LAR in three university hospitals. Different treatment approaches were compared, including a pairwise comparison of conventional treatment and endoscopic vacuum-assisted surgical closure (EVASC). Primary outcomes were healed and functional anastomosis rates at end of follow-up.

**Results:**

Overall, 103 patients were included, of which 59 underwent conventional treatment and 23 EVASC. Median number of reinterventions was 1 after conventional treatment, compared to 7 after EVASC (*p* < 0.01). Median follow-up was 39 and 25 months, respectively. Healed anastomosis rate was 61% after conventional treatment, compared to 78% after EVASC (*p* = 0.139). Functional anastomosis rate was higher after EVASC, compared to conventional treatment (78% vs. 54%, *p* = 0.045). Early initiation of EVASC in the first week after primary surgery resulted in better functional anastomosis rate compared to later initiation (100% vs. 55%, *p* = 0.008).

**Conclusion:**

Proactive treatment of AL consisting of EVASC resulted in improved healed and functional anastomosis rates for AL after LAR for rectal cancer, compared to conventional treatment. If EVASC was initiated within the first week after index surgery, a 100% functional anastomosis rate was achievable.

**Supplementary Information:**

The online version contains supplementary material available at 10.1007/s10151-023-02808-z.

## Introduction

Anastomotic leakage (AL) is one of the most dreaded complications after low anterior resection (LAR) for rectal cancer and is associated with increased rates of morbidity and mortality, higher rates of permanent stomas, worse oncological outcomes and additional healthcare costs [1–3]. The incidence of AL remains high with rates up to 20% during the first year after index surgery [4] and there is still limited evidence on the most effective treatment strategies for AL after LAR.

Conventional treatment of AL consists of the creation of a diverting ileostomy, if not created primarily, and surgical or radiological drainage of any present abscess collections. In selected patients dismantling of the anastomosis might be indicated. If initial treatment fails, an intersphincteric resection of the anastomosis with creation of an end-colostomy may be required to gain control of pelvic sepsis. More recently, proactive treatment strategies have emerged, such as endoscopic vacuum therapy (EVT). In EVT, an open-pored polyurethane sponge is placed into the presacral cavity and connected to a controlled negative pressure system, which increases local blood flow, reduces bacterial load and stimulates formation of granulation tissue. These actions lead to the gradual collapse of the abscess cavity [5, 6].

This labour-intensive protocol was first described by Weidenhagen et al. (2008) and adapted in Amsterdam, whereby vacuum therapy was only used to clean the cavity enabling surgical closure of the anastomotic dehiscence within 2 weeks—endoscopic vacuum-assisted surgical closure (EVASC) [7–9]. Previous studies showed that EVT and EVASC are effective treatments for AL, especially when initiated early after AL diagnosis in the first few weeks after the index operation [8, 10]. Other proactive treatment strategies include endoscopic clipping or transanal suturing of the defect [11].

Comparative studies on efficacy of different treatment strategies are almost non-existent in current literature. This comparative cohort study aimed to compare the efficacy of different proactive and conventional treatment strategies for AL with healed and functional anastomosis rates as primary outcomes.

## Methods

### Study population

This retrospective international multicentre cohort study included patients with AL after LAR for rectal cancer who were operated on between February 2009 and April 2020 at three university centres. Patients were excluded if they underwent surgical resection for benign disease, a partial mesorectal excision, resection without formation of an anastomosis or if they were diagnosed with a chronic sinus (leak diagnosis more than 1 year after LAR). The local medical ethical committees approved no written informed consent was necessary because of the retrospective nature of this study.

### Surgery and treatment for AL at the different centres

#### Amsterdam

In Amsterdam UMC, location AMC (AMS), patients underwent conventional total mesorectal excision (TME) with routine diversion until an institutional shift at the end of 2014 towards transanal TME (TaTME) with highly selective diversion [12]. All patients received preoperative mechanical bowel preparation and intravenous antibiotics. Throughout the entire study period, an early diagnosis and proactive treatment strategy was attained. A C-reactive protein-based imaging protocol consisting of a computed tomography (CT) scan with rectal contrast was used to diagnose AL [13]. After AL diagnosis, a diverting ileostomy was created (if not created primarily) to control pelvic sepsis and when the cavity appeared suitable, EVT was started immediately. When the cavity seemed clean with granulation tissue, it was closed with transanal sutures; 2 weeks after surgical closure, the anastomosis was evaluated endoscopically. If a healed anastomosis was observed during endoscopy and confirmed by a CT scan with rectal contrast, the diverting ileostomy was closed. A more detailed description was published earlier and a video is available on transanal closure [8, 14].

#### Oxford

In the Oxford University Hospitals (OXF), patients underwent a conventional TME or TaTME based on the operating surgeons’ preference with standard deviation. All patients received mechanical bowel preparation and intravenous antibiotics preoperatively. When there was a clinical suspicion of AL, a CT scan with rectal contrast was performed. After detection of AL, the abscess cavity was drained either surgically or endoscopically, with incidental use of transanal closure and/or EVT in more recent years, based on the surgeons’ preference. If secondary healing of the presacral cavity was achieved, and confirmed by CT scan with rectal contrast, the diverting ileostomy was closed.

#### Barcelona

In the Hospital Clinic of Barcelona (BAR), patients underwent either conventional TME or TaTME based on the operating surgeons’ preference with selective diversion. All patients received preoperative mechanical bowel preparation and intravenous antibiotics. A CT scan with rectal contrast or direct surgical intervention was performed if AL was suspected. A diverting stoma was created (if not present after LAR) and abdominal or presacral collections were drained either surgically or radiologically. After secondary healing of the presacral cavity, confirmed by CT scan with rectal contrast, the diverting stoma was closed.

### Data collection and outcome parameters

All data was retrieved from electronic medical files from the three individual hospitals and included baseline characteristics, index operation, AL diagnosis, reinterventions, readmissions and length of follow-up. Primary outcomes were healed and functional anastomosis rate. Secondary outcomes were interval from LAR to AL diagnosis, number and type of reintervention, number and reason for readmission, length of stay during index admission and related to index surgery until end of follow-up, interval from LAR to healed and functional anastomosis, type of stoma at end of follow-up and number of end colostomies.

A healed anastomosis was defined as having no active leak or chronic sinus, confirmed clinically, by endoscopy and/or by CT imaging. A functional anastomosis was defined as a healed anastomosis with restored continuity. Readmissions were counted as at least one overnight admission in the hospital. Outpatient treatment with 1-day admission (e.g. sponge exchange) was not counted as a readmission. One sponge series was defined as the period from initial placement and exchanges (including the last exchange) until any other type of intervention was performed (e.g. surgical closure) or a watch-and-wait strategy was adopted.

Conventional treatment was defined as conservative treatment with creation of a diverting stoma, if not primarily present, and drainage of present collections (either radiologically, manually or surgically) awaiting secondary healing. EVASC was defined as described above (a few rounds of EVT, followed by surgical closure of the defect), regardless of drainage or stoma creation. EVT was defined as multiple rounds of EVT without surgical closure, regardless of drainage or stoma creation. Transanal suturing was defined if only surgical closure of the defect was performed (no EVT), regardless of drainage or stoma creation. Redo-anastomosis was defined as complete dismantlement of the primary anastomosis and creation of a new secondary anastomosis. ‘Mucosal approximation strategy’ was defined as having a proactive treatment strategy in which approximation of the mucosal edges was obtained, and this included EVASC, transanal closure and redo-anastomosis.

### Statistical analysis

Results were presented separately for the different treatment strategies: conventional treatment, EVASC, EVT without surgical closure, transanal suturing and redo-anastomosis. Comparative subgroup analysis was performed for conventional vs. EVASC, early initiation of EVASC (≤ 7 days) vs. late initiation (> 7 days) and treatment including mucosal approximation vs. other treatment. Continuous data was presented as mean with standard deviation (SD) or median with interquartile range (IQR), depending on their distribution. Categorical data was presented as absolute numbers with percentages. Student’s *t* test was used for continuous and normally distributed variables. For non-normal distributed continuous variables, a Mann–Whitney *U* test was used to calculate median and IQR. Median interval in days was calculated between index operation and AL diagnosis and between index operation and first reintervention for AL. Chi-square test was used for dichotomous and categorical data. Two-sided *p* values less than 0.05 were considered statistically significant. All analyses were performed with IBM SPSS statistics, version 26.0.0 (IBM Corp. Armonk, NY, USA).

## Results

### Study population

**Fig. 1:**
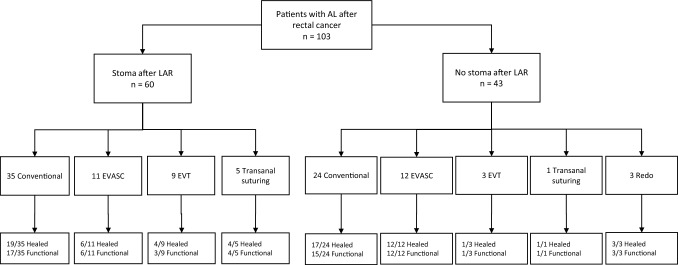
Flow diagram. *AL* anastomotic leakage, *LAR* low anterior resection, *EVASC* endoscopic vacuum-assisted surgical closure, *EVT* endoscopic vacuum therapy

A total of 103 patients were included, of whom 32 from AMS, 36 from OXF and 35 from BAR. Conventional treatment was performed in 59 patients, EVASC in 23, EVT in 12, transanal suturing in 6 and redo-anastomosis in 3. More patients had received no neoadjuvant therapy after conventional treatment (61%) and EVT (58%), compared to EVASC (39%) and transanal suturing (33%). The proportion of diverting stoma after primary resection was similar after conventional treatment (59%) and EVASC (48%), but was higher after EVT (75%) and transanal suturing (83%), see also Fig. 1. For all baseline characteristics, see Table 1.

**Table 1:** Baseline characteristics

	Conventional (*n* = 59)	EVASC (*n* = 23)	EVT (*n* = 12)	Transanal suturing (*n* = 6)	Redo (*n* = 3)
Gender (male)	45 (76%)	20 (87%)	11 (92%)	5 (83%)	2 (67%)
Mean age in years (SD)	65 (3)	64 (7)	64 (12)	59 (5)	58 (1)
Mean BMI in kg/m^2^ (SD)	26 (3)	27 (4)	26 (4)	25 (4)	25 (1)
Smoker	15 (29%)	4 (19%)	1 (14%)	1 (20%)	1 (33%)
ASA					
ASA 1	11 (19%)	4 (17%)	2 (17%)	4 (67%)	0
ASA 2	37 (63%)	13 (57%)	7 (58%)	2 (33%)	3 (100%)
ASA 3 or higher	11 (19%)	6 (26%)	3 (25%)	0	0
Neoadjuvant treatment					
None	36 (61%)	9 (39%)	7 (58%)	2 (33%)	3 (100%)
Short-course radiotherapy	3 (5%)	5 (22%)	0	1 (17%)	0
Chemoradiotherapy	19 (32%)	8 (35%)	4 (33%)	3 (50%)	0
Chemotherapy only	1 (2%)	1 (4%)	1 (8%)	0	0
Previous abdominal surgery	9 (16%)	3 (13%)	0	1 (17%)	0
Surgical approach index rectal cancer resection					
Open	1 (2%)	2 (9%)	2 (17%)	0	0
Laparoscopic	58 (98%)	21 (91%)	10 (83%)	5 (100%)	3 (100%)
Surgical technique					
LAR	30 (51%)	11 (48%)	7 (58%)	1 (17%)	0
TaTME	29 (49%)	12 (52%)	5 (42%)	5 (83%)	3 (100%)
Type of anastomosis					
Stapled	51 (93%)	21 (91%)	11 (92%)	3 (50%)	3 (100%)
Configuration					
SE	41 (70%)	15 (65%)	9 (75%)	5 (83%)	2 (67%)
EE	17 (29%)	7 (30%)	3 (25%)	1 (17%)	1 (33%)
Other	1 (2%)	1 (4%)	0	0	0
Diverting stoma after LAR					
None	24 (41%)	12 (52%)	3 (25%)	1 (17%)	3 (100%)
Created during LAR	32 (54%)	10 (44%)	8 (67%)	5 (83%)	0
Preoperative ileostomy	3 (5%)	0	0	0	0
Preoperative colostomy	0	1 (4%)	1 (4%)	0	0
Institute					
AMS	7 (12%)	19 (83%)	2 (17%)	1 (83%)	3 (100%)
OXF	18 (31%)	4 (17%)	9 (75%)	5 (83%)	0
BAR	34 (58%)	0	1 (8%)	0	0

*EVASC* endoscopic vacuum-assisted surgical closure, *EVT* endoscopic vacuum therapy, *SD* standard deviation, *BMI* body mass index, *ASA* American Society of Anesthesiologists, *LAR* low anterior resection, *TaTME* transanal total mesorectal excision, *SE* side-to-end, *EE* end-to-end, *AMS* Amsterdam, *OXF* Oxford, *BAR* Barcelona

### AL diagnosis

Timing of AL diagnosis is displayed in Table 2. AL was diagnosed within 14 days after index operation in 68% in the conventional group, compared to 78% in the EVASC group, 83% in EVT-no closure and 67% in the transanal suturing group. Differences in median interval from LAR to initiation of treatment were similar to time to diagnosis.

**Table 2:** Timing of leakage diagnosis and subsequent treatment with detailed description of reinterventions and readmissions

	Conventional (*n* = 59)	EVASC (*n* = 23)	EVT (*n* = 12)	Transanal suturing (*n* = 6)	Redo (*n* = 3)
Interval from LAR to AL diagnosis in days (IQR)	5 (3–27)	7 (4–14)	6 (2–12)	8.5 (4–19)	4 (NA)
< 14 days	40 (68%)	18 (78%)	10 (83%)	4 (67%)	3 (100%)
< 30 days	46 (78%)	20 (87%)	11 (92%)	6 (100%)	3 (100%)
< 90 days	54 (92%)	23 (100%)	11 (92%)	6 (100%)	3 (100%)
Interval from LAR to first reintervention for AL in days (IQR)	7 (4–20)	7 (4–16)	8 (3–20)	11 (4–55)	5 (NA)
Reinterventions—all					
Median (IQR)	2 (1–3)	8 (6–12)	9 (5–16)	2.5 (2–5)	2 (NA)
Reinterventions—excluding stoma creation/closure	31 (53%)	23 (100%)	12 (100%)	6 (100%)	3 (100%)
Median (IQR)	1 (0–1)	7 (5–10)	8 (4–15)	1.5 (1–4)	2 (NA)
Radiological reintervention	7 (12%)	6 (26%)	3 (25%)	0	0
Median (IQR)	0 (0–0)	0 (0–1)	0 (0–1)	NA	NA
Endoscopic reinterventions	3 (5%)	23 (100%)	12 (100%)	1 (17%)	1 (33%)
Median (IQR)	0 (0–0)	4 (3–6)	6 (3–9)	0 (0–0)	0 (NA)
Endoscopic vacuum therapy	1 (2%)	23 (100%)	12 (100%)	0	1 (33%)
Sponge series (IQR)	0 (0–0)	1 (1–1)	1 (1–1)	NA	0 (NA)
Sponge exchanges (IQR)^a^	4 (NA)	3 (2–8)	5 (3–8)	NA	3 (NA)
Surgical reinterventions—all	53 (90%)	23 (100%)	12 (100%)	6 (100%)	3 (100%)
Median (IQR)	2 (1–2)	3 (3–4)	3 (2–4)	2.5 (2–4)	2 (NA)
Surgical reinterventions—excluding stoma creation/closure	26 (44%)	23 (100%)	11 (92%)	6 (100%)	3 (100%)
Median (IQR)	0 (0–1)	1 (1–3)	2 (2–3)	1.5 (1–3)	1 (NA)
Surgical drainage	26 (44%)	5 (22%)	6 (50%)	3 (50%)	0
Median (IQR)	0 (0–1)	0 (0–0)	1 (0–1)	0.5 (0–1)	0 (0–0)
Washout	6 (10%)	7 (30%)	7 (58%)	3 (50%)	0
Median (IQR)	0 (0–0)	0 (0–1)	1 (0–2)	0.5 (0–1)	0 (0–0)
Other (e.g. rectal catheter debridement fistula etc.)	14 (24%)	5 (22%)	10 (83%)	3 (50%)	1 (33%)
Median (IQR)	0 (0–0)	0 (0–0)	1 (1–1)	0.5 (0–2)	0 (NA)
Transanal closure	5 (9%)	23 (100%)	3 (25%)^b^	6 (100%)	0
Median (IQR)	0 (0–0)	1 (1–2)	0 (0–1)	1 (1–1)	0 (0–0)
Redo-anastomosis	–	–	–	–	3 (100%)
Median (IQR)	0 (0–0)	0 (0–0)	0 (0–0)	0 (0–0)	1 (1–1)
Resection anastomosis with end-colostomy	14 (24%)	4 (17%)	4 (33%)	1 (17%)	0
Median (IQR)	0 (0–0)	0 (0–0)	0 (0–1)	0 (0–0)	0 (0–0)
Stoma-related surgical reinterventions					
Creation/correction of stoma	19 (31%)	14 (71%)	4 (33%)	1 (17%)	3 (100%)
Median (IQR)	0 (0–1)	1 (0–1)	0 (0–1)	0 (0–0)	1 (1–1)
Ileostomy reversal	38 (64%)	22 (96%)	5 (42%)	6 (100%)	3 (100%)
Median (IQR)	1 (0–1)	1 (1–1)	0 (0–1)	1 (1–1)	1 (1–1)
Readmissions	50 (85%)	23 (100%)	9 (75%)	6 (100%)	3 (100%)
Median (IQR)	1 (1–2)	2 (1–4)	2 (0–3)	3 (2–4)	2 (NA)
Readmissions—excluding stoma creation/closure	32 (54%)	16 (70%)	9 (75%)	5 (83%)	2 (56%)
Median (IQR)	1 (0–1)	1 (0–3)	2 (0–3)	2 (1–3)	1 (NA)
Treatment for AL	17 (29%)	14 (61%)	8 (67%)	3 (50%)	1 (33%)
Median (IQR)	0 (0–1)	1 (0–2)	1 (0–1)	1 (0–2)	0 (NA)
Ileus	5 (9%)	1 (4%)	2 (33%)	2 (33%)	0
Median (IQR)	0 (0–0)	0 (0–0)	0 (0–1)	0 (0–1)	0 (0–0)
Other	9 (15%)	3 (13%)	2 (17%)	2 (33%)	1 (33%)
Median (IQR)	0 (0–0)	0 (0–0)	0 (0–0)	0 (0–1)	0 (NA)
Stoma-related readmissions					
Stoma closure	35 (59%)	20 (87%)	2 (17%)	56 (100%)	3 (100%)
Median (IQR)	1 (0–1)	1 (1–1)	0 (0–0)	1 (1–1)	1 (1–1)
Stoma-related problems	6 (10%)	4 (17%)	0	2 (33%)	0
Median (IQR)	0 (0–0)	0 (0–0)	0 (0–0)	0 (0–1)	0 (0–0)
Length of stay					
Index admission for LAR in days (IQR)	12 (7–20)	15 (5–25)	30 (18–58)	10 (5–31)	10 (NA)
During complete FU in days (IQR)	19 (12–31)	30 (23–43)	52 (33–88)	22 (13–49)	13 (NA)
Total—without stoma closure in days (IQR)	16 (9–28)	25 (19–34)	52 (33–84)	18 (10–37)	11 (NA)

*EVASC* endoscopic vacuum-assisted surgical closure, *EVT* endoscopic vacuum therapy, *LAR* low anterior resection, *AL* anastomotic leakage, *IQR* interquartile range, *FU* follow-up, *NA* not available

^a^Only patients who underwent EVT were included in this analysis

^b^A total of three patients underwent surgical closure before start of endoscopic vacuum therapy

### Reinterventions

Reintervention rate excluding stoma creation/closure was only 53% in the conventional group, compared to 100% in the other groups. The median number of reinterventions was highest in the EVT group (8, IQR 4–15), followed by EVASC (7, IQR 5–10), transanal suturing (1.5, IQR 1–4) and conventional treatment (1, IQR 0–1).

Resection of the anastomosis with creation of end-colostomy was performed most often in the EVT group (33%), followed by conventional treatment (24%), transanal suturing (17%) and EVASC (17%).

### Readmissions

Readmission excluding stoma creation/closure was highest in transanal suturing (83%), followed by EVT (75%), EVASC (70%) and conventional treatment (54%). Median number of readmissions was higher in EVT and transanal suturing (2, IQR 0–3 and 2, IQR 1–3), compared to conventional and EVASC (1, IQR 0–1 and 1, IQR 0–3). Reasons for readmission were mainly for treatment of AL. Median total length of stay excluding stoma closure/creation was highest after EVT (52 days, IQR 33–84), followed by EVASC (25 days, IQR 19–34), transanal suturing (18 days, IQR 10–37) and conventional treatment (16 days, IQR 9–28).

### Surgical outcomes

The outcomes regarding anastomotic healing and bowel continuity after a median follow-up of 25–39 months are displayed in Table 3. The percentage of healed anastomosis at the end of follow-up was 61% after conventional treatment, 78% after EVASC, 42% after EVT and 83% after transanal suturing.

**Table 3:** Surgical outcomes

	Conventional (*n* = 59)	EVASC (*n* = 23)	EVT (*n* = 12)	Transanal suturing (*n* = 6)	Redo (*n* = 3)
Median follow-up in months (IQR)	39 (24–62)	25 (12–59)	30 (21–61)	37 (32–45)	19 (NA)
Healed anastomosis at EFU	36 (61%)	18 (78%)	5 (42%)	5 (83%)	3 (100%)
Median interval from LAR to healed anastomosis in days (IQR)	141 (77–216)	114 (48–210)	304 (197–567)	104 (60–252)	153 (NA)
Functional anastomosis at EFU	32 (54%)	18 (78%)	4 (33%)	5 (83%)	3 (100%)
Median interval from LAR to functional anastomosis in days (IQR)	267 (142–368)	185 (146–292)	364 (325–676)	296 (207–353)	188 (NA)
Stoma at EFU					
Pre-LAR ileostomy	2 (3%)	0	0	0	0
Primary ileostomy (created during LAR)	4 (7%)	1 (4%)	4 (33%)	0	0
Secondary ileostomy (after LAR)	2 (3%)	0	0	0	0
Tertiary ileostomy (stoma after stoma closure)	1 (2%)	0	1 (8%)	0	0
End-colostomy	14 (24%)	4 (17%)	3 (25%)	1 (17%)	0
No stoma, not healed at EFU	4 (7%)	0	0	0	0

*EVASC* endoscopic vacuum-assisted surgical closure, *EVT* endoscopic vacuum therapy, *LAR* low anterior resection, *AL* anastomotic leakage, *IQR* interquartile range, *EFU* end of follow-up, *NA* not available

Median interval from LAR to healed anastomosis was shortest after transanal suturing (104 days, IQR 60–252), followed by EVASC (114 days, IQR 48–210), conventional treatment (141 days, IQR 77–216) and EVT (304 days, 197–567).

The highest proportion of patients with a functional anastomosis was found for transanal suturing (83%), followed by EVASC (78%), conventional treatment (54%) and EVT (33%). Median interval from LAR to functional anastomosis was shortest in EVASC (185 days, IQR 146–292), compared to conventional (267 days, IQR 142–368), transanal suturing (296 days, IQR 207–353) and EVT (364 days, IQR 325–676).

### Pairwise comparison and subgroup analysis

Pairwise comparison showed a higher healed anastomosis rate after EVASC compared to conventional treatment (78% vs. 61%), although this was not statistically significant (*p* = 0.139) (Table 4). The functional anastomosis rate was significantly higher after EVASC, when compared to conventional treatment (78% vs. 54%, *p* = 0.045). In the EVASC group more surgical reinterventions were performed (median 3 vs. 2, *p* < 0.001), more readmissions (median 2 vs. 1, *p* < 0.001) and a longer length of stay (median 30 vs. 19 days, *p* = 0.004) were seen, compared to conventional treatment. More planned readmissions were seen after EVASC (median 1 vs. 2, *p* < 0.001), but no difference in unplanned readmissions was seen (median 0 vs. 0, *p* = 0.479).

**Table 4:** Pairwise comparison and subgroup analysis

	Conventional (*n* = 59)	EVASC (*n* = 23)	*P* value
Median follow-up in months (IQR)	39 (24–62)	25 (12–19)	0.124
Interval LAR–AL diagnosis	5 (3–27)	7 (4–14)	0.921
Interval LAR–first reintervention for AL	7 (4–20)	7 (4–16)	0.918
Healed anastomosis at EFU	36 (61%)	18 (78%)	0.139
Median interval from LAR to healed anastomosis in days (IQR)	141 (77–216)	114 (48–210)	0.271
Functional anastomosis at EFU	32 (54%)	18 (78%)	0.045
Median interval from LAR to functional anastomosis in days (IQR)	267 (142–368)	185 (146–292)	0.245
Median number of reinterventions	2 (1–3)	8 (6–12)	< 0.001
Median number of surgical reinterventions	2 (1–2)	3 (3–4)	< 0.001
Median number of readmissions	1 (1–2)	2 (1–4)	< 0.001
Planned readmissions	1 (0–1)	2 (1–2)	< 0.001
Unplanned readmissions	0 (0–1)	0 (0–1)	0.479
Total length of stay	19 (12–31)	30 (23–43)	0.004

	EVASC (*n* = 23)		*p* value
	Early (≤ 7 days) (*n* = 12)	Late (> 7 days) (*n* = 11)	
Healed anastomosis at EFU	12 (100%)	6 (55%)	0.008
Median interval from LAR to healed anastomosis in days (IQR)	107 (44–185)	123 (90–357)	0.291
Functional anastomosis at EFU	12 (100%)	6 (55%)	0.008
Median interval from LAR to functional anastomosis in days (IQR)	185 (128–258)	233 (152–393)	0.250
Median number of reinterventions	9 (6–12)	8 (6–13)	0.880
Median number of readmissions	1 (1–2)	4 (2–7)	< 0.001
Total length of stay	29 (23–39)	30 (18–60)	0.566

	Mucosal approximation^a^ (*n* = 32)	Passive/other (*n* = 71)	*p* value
Healed anastomosis at EFU	26 (81%)	41 (58%)	0.021
Median interval from LAR to healed anastomosis in days (IQR)	114 (64–204)	163 (80–248)	0.080
Functional anastomosis at EFU	26 (81%)	36 (51%)	0.003
Median interval from LAR to functional anastomosis in days (IQR)	207 (151–298)	291 (148–371)	0.138
Median number of reinterventions	8 (3–10)	2 (1–3)	< 0.001
Median number of readmissions	2 (1–3)	1 (1–2)	< 0.001
Total length of stay	29 (18–42)	23 (15–46)	0.237

*EVASC* endoscopic vacuum-assisted surgical closure, *IQR* interquartile range, *LAR* low anterior resection, *AL* anastomotic leakage, *EFU* end of follow-up

^a^Mucosal approximation was defined as having a proactive treatment strategy in which approximation of the mucosal edges was obtained, and this included EVASC, transanal closure and redo-anastomosis

If EVASC was started in the first 7 days after surgery, the healed anastomosis rate was higher (100% vs. 55%, *p* = 0.008), compared to late initiation (> 7 days). Similarly, the functional anastomosis rate was higher (100% vs. 55%), with similar median number of reinterventions (9 vs. 8, *p* = 0.880) and length of stay (29 vs. 30 days, *p* = 0.566), but fewer readmissions (1 vs. 4, *p* < 0.001).

If mucosal approximation was obtained this led to higher healed anastomosis rate (81% vs. 58%, *p* = 0.021) and higher functional anastomosis rates (81% vs. 51%, *p* = 0.003), when compared to passive closure or other treatments. Median numbers of reinterventions (8 vs. 2, *p* < 0.001) and readmissions (2 vs. 1, *p* < 0.001) were higher after mucosal approximation, compared to passive closure or other treatments.

## Discussion

This three-centre international comparative cohort study shows that proactive treatment of AL that aims to achieve mucosal approximation leads to better healed and functional anastomosis rates. These improved outcomes of a proactive strategy, however, require the highest number of reinterventions and readmissions. Subgroup analysis showed that EVASC leads to better results than conventional treatment and that a 100% success rate in achievable if EVASC is started in the first week after surgery. These findings warrant further explorative studies to define the most optimal treatment strategy for AL.

A systematic review on EVT for AL reported a stoma reversal rate of 75.9%, which is comparable to the results seen after EVASC [6]. However, AL is a heterogeneous disease entity and successful treatment depends on multiple factors. This complicates direct comparisons between published series. A recent prospective cohort study from the GRECCAR group showed an overall success rate of 55%, and this was 72% if treatment was started within 15 days after index operation and 28% beyond 15 days [10]. A Dutch population-based study showed that applied treatments for AL after LAR in routine daily practice in 2011 were successful in 52%, resulting in a chronic sinus rate of 9.5% for the total cohort of patients who underwent resection for rectal cancer [8]. A French single-centre study that investigated the efficacy of radiological or transanal drainage showed success in 50% of patients after initial treatment [15]. At end of follow-up, 80% of patients were stoma-free, but many patients required major salvage surgery by performing a redo-anastomosis (39%). Studies that compare proactive with conventional treatment strategies for AL after LAR are scarce. Kühn et al. compared EVT treatment with a historical cohort that underwent conventional treatment, and found higher restored continuity rates after EVT treatment (86.7 vs. 37.5%, *p* = 0.001) [16]. Similar to our results, they found a shorter length of stay after conventional treatment (31 vs. 42 days), but time to stoma closure was not different.

One of controversial topics is the creation of a primary diverting stoma after LAR. More selective diversion (AMS and BAR) has all the advantage of not having a stoma in the majority of patients who will never develop AL, but does requires construction of secondary diverting stoma in case of AL. Opponents of a selective approach emphasize the risk of losing anastomotic integrity in the end, but the present data actually shows that routine diversion does not increase the chance of bowel continuity in case of AL. Furthermore, although a diverting ileostomy is created with a temporary intention, up to 28% of them eventually become a permanent stoma [17]. Interestingly, permanent stomas consisted of colostomies in the vast majority of patients after selective diversion (AMS and BAR), while these were often the initial ileostomy after routine diversion (OXF).

Although EVASC required more planned readmissions and a longer length of stay, compared to conventional treatment, we believe the impact for the patient was less severe than the results suggest. Most reinterventions and readmissions for sponge replacement, transanal closure or stoma closure could be planned and performed in a controlled setting. No differences in unplanned readmissions was seen and the actual moment AL was diagnosed often occurred during the primary admission, saving a visit to the emergency ward.

Functional outcomes were not measured in this cohort series because of the retrospective study design, but could have been more favourable in the proactive treatment group. The development of low anterior resection syndrome (LARS) is multifactorial, including postoperative changes of the pelvic floor and sphincter function, height of the anastomosis, neoadjuvant treatment and alterations in colonic microbiota [18, 19]. Another factor has been analysed in a recent meta-analysis, which showed an increased risk of majors LARS in patients with an ileostomy (OR 2.84, 95% CI 1.70–4.75) and in patients with a longer time to stoma closure (mean difference 2.39 months, OR 1.28–3.51) [20]. Proactive treatment of AL resulted in restored continuity almost 5 months earlier in AMS compared to the other two centres.

This study has a number of limitations. First, there are several methodological issues related to the retrospective study design and relatively small sample size. Although the retrospective nature could lead to potential loss of data, all data regarding the primary outcomes was complete in this study. Second, the definition of a healed anastomosis is debatable. Regular endoscopic control of the anastomosis was performed in AMS, but not in the other centres. Because of restricted accuracy of endoscopy and imaging, a ‘healed’ anastomosis might still hide a small sinus behind it, which might become active if restoring bowel continuity. Therefore, these results should be interpreted with caution. It should be noted that the functional anastomosis rate might be more valuable as an outcome compared to the healed anastomosis rate, because it represents a more reliable and relevant outcome. Third, there might be performance bias by including patients from different hospitals. However, this seems to be the only feasible way to compare proactive and conventional treatment for AL at present because of the practical implications. Fourth, no details on leak size were available. It is possible that the high success rate in the transanal closure group might be because at the time of diagnosis the defect was limited and direct surgical closure was feasible. Fifth, differences in rates of neoadjuvant treatment and diverting stoma after primary surgery were seen between groups, which may influence success rates. Finally, larger cohort series are needed to confirm the present findings and to increase insight into the most effective treatment modalities (e.g. TENTACLE study [21]).

## Conclusion

A proactive treatment strategy consisting of EVASC resulted in a higher healed and functional anastomosis rate, compared to conventional treatment, and a 100% success rate is achievable if EVASC is initiated within the first week after primary surgery. This could justify the need for a higher number of reinterventions if applying a proactive treatment.

### Supplementary Information

Below is the link to the electronic supplementary material.

Supplementary file1 (DOCX 23 KB)

## Data Availability

Data will be available on reasonable request to the authors.
