# Supplementary material for: Comparison of proactive and conventional treatment of anastomotic leakage in rectal cancer surgery: a multicentre retrospective cohort series
Source: Tech Coloproctol. 2023 May 22;27(11):1099–108. doi: 10.1007/s10151-023-02808-z (PMC10562258; doi:10.1007/s10151-023-02808-z)
Supplement: Supplementary file 1 — Supplementary file1 (DOCX 23 KB) [file 10151_2023_2808_MOESM1_ESM.docx]

Supplementary table 1: Oncological staging

|  | **Conventional**  **(n=59)** | **EVASC**  **(n=23)** | **EVT**  **(n=12)** | **Transanal suturing**  **(n=6)** | **Redo**  **(n=3)** |
| --- | --- | --- | --- | --- | --- |
| **Pathological stage** |  |  |  |  |  |
| T-stage |  |  |  |  |  |
| T0 | 1 (2%) | 1 (4%) | 0 | 1 (17%) | 0 |
| T1 | 6 (10%) | 4 (17%) | 2 (17%) | 1 (17%) | 0 |
| T2 | 20 (34%) | 8 (35%) | 1 (8%) | 1 (17%) | 0 |
| T3 | 30 (51%) | 8 (35%) | 8 (67%) | 3 (50%) | 3 (100%) |
| T4 | 2 (3%) | 2 (9%) | 1 (8%) | 0 | 0 |
| N-stage |  |  |  |  |  |
| N1 or higher | 19 (32%) | 8 (35%) | 4 (33%) | 2 (33%) | 0 |
| M-stage |  |  |  |  |  |
| M1 | 3 (5%) | 5 (22%) | 0 | 2 (33%) | 0 |

**Supplementary table 2**. Primary outcomes compared between centers

|  | **AMS vs OXF** | | **AMS vs BAR** | | **BAR vs OXF** | |
| --- | --- | --- | --- | --- | --- | --- |
|  | Absolute diff. | P-value | Absolute diff. | P-value | Absolute diff. | P-value |
| **Healed anastomosis** | +8% | 0.482 | +11.9% | 0.307 | -3.9% | 0.736 |
| **Median Interval from LAR to healed anastomosis** | -102 days | 0.000 | -56 days | 0.023 | -46 days | 0.118 |
| **Functional anastomosis** | +13.6% | 0.243 | +20.5% | 0.086 | -6.9% | 0.559 |
| **Median Interval from LAR to functional anastomosis** | -144 days | 0.000 | -164 days | 0.008 | +20 days | 0.899 |

*AMS: Amsterdam; OXF: Oxford; BAR: Barcelona; diff: difference; LAR: low anterior resection;*
